# Repetitive Transcranial Magnetic Stimulation for Comorbid Major Depressive Disorder and Alcohol Use Disorder

**DOI:** 10.3390/brainsci12010048

**Published:** 2021-12-30

**Authors:** Victor M. Tang, Bernard Le Foll, Daniel M. Blumberger, Daphne Voineskos

**Affiliations:** 1 Temerty Centre for Therapeutic Brain Intervention, Centre for Addiction and Mental Health, 1025 Queen St. W, Toronto, ON M6J 1H1, Canada; daniel.blumberger@camh.ca (D.M.B.); daphne.voineskos@camh.ca (D.V.); 2 Translational Addiction Research Laboratory, Centre for Addiction and Mental Health, 33 Ursula Franklin St., Toronto, ON M5S 2S1, Canada; bernard.lefoll@camh.ca; 3 Department of Psychiatry, Temerty Faculty of Medicine, University of Toronto, 250 College St., Toronto, ON M5T 1R8, Canada; 4 Institute of Medical Sciences, Temerty Faculty of Medicine, University of Toronto, 1 King’s College Circle, Toronto, ON M5S 1A8, Canada; 5 Campbell Family Mental Health Research Institute, Centre for Addiction and Mental Health, 250 College St., Toronto, ON M5T 1R8, Canada; 6 Department of Pharmacology & Toxicology, Temerty Faculty of Medicine, University of Toronto, 27 King’s College Circle, Toronto, ON M5S 3H7, Canada; 7 Department of Family and Community Medicine, Temerty Faculty of Medicine, University of Toronto, 500 University Ave, Toronto, ON M5G 1V7, Canada

**Keywords:** transcranial magnetic stimulation, alcohol use disorder, major depressive disorder, comorbidity

## Abstract

Major depressive disorder (MDD) and alcohol use disorder (AUD) are leading causes of disability, and patients are frequently affected by both conditions. This comorbidity is known to confer worse outcomes and greater illness severity. Repetitive transcranial magnetic stimulation (rTMS) is a non-invasive neuromodulation method that has demonstrated antidepressant effects. However, the study of rTMS for patients with MDD and commonly associated comorbidities, such as AUD, has been largely overlooked, despite significant overlap in clinical presentation and neurobiological mechanisms. This narrative review aims to highlight the interrelated aspects of the literature on rTMS for MDD and rTMS for AUD. First, we summarize the available evidence on the effectiveness of rTMS for each condition, both most studied through stimulation of the dorsolateral prefrontal cortex (DLPFC). Second, we describe common symptom constructs that can be modulated by rTMS, such as executive dysfunction, that are transdiagnostic across these disorders. Lastly, we describe promising approaches in the personalization and optimization of rTMS that may be applicable to both AUD and MDD. By bridging the gap between research efforts in MDD and AUD, rTMS is well positioned to be developed as a treatment for the many patients who have both conditions concurrently.

## 1. Introduction

Major depressive disorder (MDD) is highly prevalent in individuals with alcohol use disorder (AUD) [1]. The National Epidemiologic Survey on Alcohol and Related Conditions found that in those with lifetime MDD, 40% had any lifetime AUD [2]. This comorbidity also increases the complexity and burden of illness of either alone, due to increased risk of suicide [3], length and recurrence of depressive illness [4], presence of other psychiatric comorbidities [5], and greater health service utilization [6]. Despite the need for effective treatments in comorbid MDD and AUD (MDD + AUD), current options are very limited. Although randomized controlled trials and meta-analyses have shown efficacy for antidepressants in MDD with comorbid AUD [7,8], the effects are modest, with a mix of positive and negative studies [8], with a recent Cochrane review showing no effect when excluding studies with a high risk of bias [7]. Similarly, studies for treatments to reduce alcohol use in MDD + AUD remain very limited and without clear effect [9]. Approved AUD medications, such as naltrexone, have been studied in combination with antidepressants with mixed results [10,11,12]. Moreover, certain medications, when combined with excessive alcohol use, may result in added safety risks, such as in the case of bupropion, which may lower the individual’s seizure threshold. Ref. [13] The current standard of care for those with comorbid MDD + AUD remains the use of evidence-based, disorder-specific treatments for each MDD and AUD [14]. While these MDD-specific and AUD-specific treatments may be offered sequentially, in parallel, or integrated, ultimately, treatments for AUD and treatments for MDD are separate and distinct. Little work has been done to develop therapeutic approaches directly targeting the intersection of both disorders. A growing understanding of the neurobiological mechanisms of both MDD and AUD may suggest potential treatment targets to treat the comorbidity directly and could lead to more optimal outcomes than the conventional approach of treating these conditions separately.

Therapeutic non-invasive neuromodulation is an increasingly promising frontier in our efforts to develop brain-based approaches to the development of novel therapeutics and may be a promising treatment strategy for the treatment of comorbid conditions. Of these, repetitive transcranial magnetic stimulation (rTMS) is a particularly exciting modality, given the rapidly growing evidence base [15] and theoretical advantages, such as the ability to stimulate discrete and focal regions of the brain implicated in the pathophysiology of neuropsychiatric disorders [16]. rTMS operates by generating a rapidly alternating electric current within a coil that is placed over the scalp, inducing a magnetic field in a localized region of the brain below and creating an electrical current in the brain through neuronal depolarization. This is best exemplified through studies of the primary motor system, where a single pulse of TMS to the motor cortex results in a motor-evoked potential measured by electromyography and muscle contraction observed on visual inspection. When these TMS pulses are delivered repetitively (i.e., rTMS), particularly over multiple sessions across multiple days, durable neuroplastic changes can be observed through functional or structural biomarkers [17], commonly through studies of magnetic resonance imaging (MRI) or electroencephalography (EEG), before and after rTMS. Such changes are observable not only directly at sites of stimulation, but also across distributed networks related to the initial target site [18]. Through focal modulation of selected regions of the brain, the induced changes on neuronal excitation or inhibition have effects on ameliorating mood and addiction-related mechanisms. Illustrative of this are studies showing that rTMS to prefrontal circuits implicated in emotional learning and memory can disrupt learned fear responses [19], which has broad implications for many of the mood and anxiety symptoms we see clinically [20]. Research into the neuromodulation of neural circuits involved in the acquisition and consolidation of emotional events demonstrates how TMS can be used as both an investigational and clinical tool [21]. Currently, rTMS is used most in psychiatry for the treatment of MDD, particularly in those unable to benefit from initial trials of antidepressant medication [22,23].

The potential of rTMS as a treatment for MDD is established, and for AUD, it is beginning to be realized; however, we propose that it may also be a promising avenue for the treatment of comorbidity between these disorders as well. The current standard in the field is to study these disorders separately. Trials of rTMS for MDD are typically designed to exclude those with comorbid AUD, and vice versa. We describe the very small number of rTMS studies that report on treatment effects in patients with AUD and comorbid depression, dysthymia, or depressive symptoms. However, in general, rTMS for MDD + AUD remains a relatively unexplored topic. In this narrative review, we outline the evidence to date from clinical trials of rTMS in MDD and AUD individually and highlight points of overlap in the literature that suggest how rTMS may be more directly tailored to these conditions when they are comorbid. We further this proposal with a discussion of how rTMS can address comorbidity in general by targeting transdiagnostic symptom constructs that can be informed by our understanding of the neurobiology, and specific targets can be selected based on clinical relevance and importance. Lastly, we explore some of the current frontiers of rTMS research that aim to optimize and individualize treatment outcomes, again with a focus on findings that appear to overlap in MDD and AUD.

## 2. Evidence for rTMS in MDD

For psychiatric disorders, rTMS is most rigorously studied in MDD [24]. After the early foundational studies of TMS on the motor cortex, rTMS soon became feasible and demonstrated antidepressant effects in early case reports [25] and preclinical models [26]. Based on research implicating the dorsolateral prefrontal cortex (DLPFC) in the pathophysiology of MDD, early pilot studies of rTMS targeting this region yielded promising results [27]. This provided the rationale for seminal trials in the field that showed rTMS to the left DLPFC was effective for MDD [28,29], which resulted in Health Canada (2004) and Food and Drug Administration (2008) approval. The standard protocol typically involves high frequency stimulation (HF) at an intensity above the individual’s motor threshold. Other parameters that are supported by high quality evidence include low frequency (LF; 1 Hz) rTMS to the right DLPFC, and bilateral LF right followed by HF left DLPFC rTMS. Typically, HF stimulation (5–20 Hz) is considered to be excitatory and low frequency stimulation (1–5 Hz) to be inhibitory [30]. It has been posited that these DLPFC treatments ameliorate functional asymmetry, i.e., left hypoactivity and right hyperactivity [31,32]. Head-to-head studies and network meta-analyses have not convincingly demonstrated the superiority of one approach over the other [33,34], and clinical differences may be found in the better tolerability of LF-rTMS and differences in treatment time [35]. Another successful approach is to expand the site of stimulation with deep TMS (dTMS), using H-coils specially designed to reach greater depths (up to 4–5 cm) with less focality [36]. This allows for stimulation of the DLPFC bilaterally (but preferentially to the left) and beyond into the general PFC region, including the medial prefrontal cortex (MPFC), and some stimulation of deeper structures, such as the amygdala, hippocampus, and thalamus [37]. Developers of this coil have also suggested the ability of deeper PFC stimulation to yield stronger secondary activation of limbic structures, compared to stimulation of superficial sites, such as the DLPFC [38]. After large, multi-site, double-blind RCTs demonstrated the efficacy of the H1 dTMS coil [39], FDA approval was established in 2013 for MDD. Together with the support of clinical practice guidelines [40], international consensus recommendations [15], and meta-analyses [41], these aforementioned protocols are now established treatments for MDD. Given that the majority of these trials were done in those with some degree of treatment resistance to antidepressant pharmacotherapy, these recommendations suggest that first-line evidence is available specifically in those who have inadequate response to ≥1 adequate trial of an antidepressant in the current depressive episode.

The most recent pivotal development in rTMS for MDD is the development of theta burst stimulation (TBS). TBS provides the opportunity to treat people with a faster and more efficient protocol, given that it can be completed in a fraction of the time, compared to standard rTMS, by using a pattern of stimulation that mimics endogenous theta rhythms that induce plasticity in hippocampal brain slices of rodents [42]. Adapted to rTMS machines, TBS stimulates the cortex with triplet pulses of rTMS applied at 50 Hz (burst) and delivered every 200 msec (5 Hz). The original TBS paradigms applied continuous TBS (cTBS) involving a 40 s uninterrupted train (i.e., 600 pulses) while intermittent TBS (iTBS) involves a 2 s train of bursts repeated every 8 s for a total of 600 pulses [43], although modifications to this protocol have been studied since then. The cTBS protocol was found to decrease cortical excitability and iTBS to increase cortical excitability [43]. It was proposed that TBS may produce enduring neuroplastic changes through synaptic mechanisms of long-term depression in cTBS and long-term potentiation in iTBS [42]. A pivotal multi-site RCT of iTBS to the DLPFC, resulting in FDA approval, demonstrated that iTBS was found to be non-inferior to standard HF-rTMS in MDD [44]. A single 600 pulse iTBS session is 3 min, compared to 37.5 min in HF-rTMS. Although there are many more years of evidence accumulated for HF-rTMS [15], iTBS is poised to become one of the predominant rTMS modalities, given the ability to vastly increase efficiency and decrease costs in routine clinical practice. Beyond these rTMS treatments, there has been a vast proliferation of studies looking into other protocols that explore the differential effects of frequency, pattern, pulse number, treatment number, and so on. Indeed, it was estimated that there are >360 clinical studies of rTMS for MDD [45]. These have included modest or emerging levels of evidence into double cone coils that preferentially target the MPFC [46], priming TMS using low frequency stimulation followed by high frequency stimulation [47], and accelerated protocols, where multiple rTMS sessions are delivered daily [48].

## 3. Evidence for rTMS in AUD

The literature on rTMS for the treatment of AUD is less mature, with considerably fewer studies and lower-level evidence compared to MDD [24]. A summary of clinical trials included in this review is provided in Table 1. Clinical research into rTMS for other substances of abuse preceded studies for AUD, beginning with nicotine [49] and cocaine [50]. In 2010, the first clinical trial in AUD was published, a single-blind, sham-controlled RCT of 45 patients that showed significant reductions in alcohol craving following 10 sessions of active right HF-rTMS (10 Hz, 1000 pulses per treatment) compared to sham [51]. From this study, other trials of HF-rTMS to the right DLPFC were conducted, although the majority delivered only 1–2 treatment sessions as the intervention [52,53,54,55] and resulted in no significant effect in reducing alcohol cravings. Another pair of studies represented an overlapping patient sample and delivered 14–15 HF-rTMS to the right DLPFC at 20 Hz and 1560 pulses per treatment [56,57]. These authors found that general, but not cue-induced, cravings decreased with treatment; however, there was no sham comparator group. The use of HF-rTMS on the right DLPFC is different than what is commonly targeted in MDD, which is the left DLPFC. The rationale for stimulating right DLPFC in the initial report [51] was due to the superiority of right over left stimulation in early studies of cocaine dependence [50]. Furthermore, there are studies that show that the right DLPFC is preferentially activated in cognitive control processes inhibiting cue-induced craving and drug seeking [52]. Corroborating this is a small study of inhibitory cTBS delivered to the right DLPFC, leading to increases in ad libitum alcohol consumption in a controlled experimental paradigm and decrease in performance on the stop-signal reaction time task, which measures inhibition [58]. For left-sided HF-rTMS, two small RCTs are available, both showing no significant differences between active and control conditions on alcohol craving [59,60]. However, these studies are limited by quite small sample sizes (*n* = 19 and 17, respectively), and the number of sessions in the treatment course are low (10 and 4, respectively). Interestingly, in a small trial of 20 patients randomized to either right or left DLPFC HF-rTMS, both groups showed decreases in craving but with no difference between groups [61].

More consistent benefit was seen, using dTMS. Using the H1 dTMS coil, two small RCTs showed significant decreases in symptoms of AUD in active compared to sham [62,65], and an open-label, nonrandomized trial of adjunctive dTMS vs. standard drug treatment showed superiority of dTMS in reducing symptoms of craving and depression [63]. As mentioned previously, the H1 coil provides stimulation to deep prefrontal regions bilaterally, but preferentially to the left. A single group open label trial with a subset of patients with comorbid MDD and AUD also showed benefit on craving [64]. A follow up study of this open label trial showed significant decreases in depression symptoms of 21 patients with comorbid MDD and AUD [70]. Interestingly, however, a recent large double-blind RCT of 54 AUD patients receiving 15 daily treatments with the H8 dTMS coil at 10 Hz and 1500 pulses per session or sham did not show any significant differences between groups on alcohol craving or consumption [69]. The H8 coil is different than the aforementioned H1 coil, as it is designed to specifically target the insula and does not stimulate the anterior prefrontal areas and is a bilateral coil. The insula was hypothesized to be a promising target, due to it being an important region in interoception and integrating internal states and strongly associated with craving [71]. Furthermore, this stimulation protocol was intended to parallel the protocol that was shown to be effective in smoking cessation [72,73]; however, these smoking trials used an H4 coil, which stimulated not only the insula, but also the bilateral prefrontal (which the H8 coil does not). Lastly, two studies of single-session cTBS to a total of 3600 pulses to the MPFC demonstrated the ability to modify fronto-striatal activity on functional MRI but not on self-reported craving [66,67], and requires further study with a full course of treatment.

## 4. Developing rTMS to Treat MDD and AUD Comorbidity

The summary of evidence demonstrates that rTMS for MDD is an established and effective treatment option, and for AUD, there is accumulating pilot data, which in conjunction with evidence showing rTMS effectiveness in other SUDs [74], has great potential for also being a therapeutic option in the future. Treatment of patients with comorbid MDD + AUD with rTMS may require a unique approach that arises from the synthesis of the separate bodies of evidence that exist for each individual disorder (Table 2). It is worth noting, though, that several limitations exist in extrapolating the results of pilot clinical trials in AUD. One issue is the common occurrence of studies only assessing craving after one rTMS treatment session. In MDD, therapeutic effects are not expected after one session, and indeed meta-analyses have shown that a greater number of sessions is associated with increased efficacy [75], and this was shown in craving for substances as well [76]. Furthermore, the current standard for an adequate course of treatment is 20–30 sessions [30]. To our knowledge, only 2 studies have looked at 20 session courses [63,64] and 2 studies looked at 15 sessions [56,69], with only one study from each these having control groups [63,69]. Another limitation is the focus on craving as the primary outcome, with much fewer studies assessing the effects on actual consumption. Lastly, we note that there are very few trials focused on using rTMS to treat comorbid MDD + AUD. One group of authors reported on rTMS for MDD + AUD in two publications with an overlapping sample of patients [64,70], and one reported on comorbid dysthymia and AUD [63]. Otherwise, studies of rTMS are known to specifically target MDD or AUD exclusive of the other disorder.

Excitatory stimulation of the left DLPFC was confirmed to be effective for MDD; however there was not a significant signal in pilot RCTs for AUD. This suggests that excitatory left DLPFC stimulation is more effective for depression than AUD, and indeed, Del Felice and colleagues [60] showed that HF-rTMS over the left DLPFC decreased symptoms of depression (in patients without a diagnosis of MDD) but had no effect on alcohol cravings. In AUD, there is only literature exploring the efficacy of excitatory stimulation in the right DLPFC; however, for MDD, mostly inhibitory stimulation was studied here. In AUD, one study of inhibitory stimulation using cTBS to the right DLPFC actually found increases in a lab-based measure of alcohol consumption [58]. Although study results for right DLPFC HF-rTMS are mixed, the largest and highest quality trial of these did show a significant effect on alcohol craving [51]. There is scarce evidence for HF-rTMS to the right DLPFC in MDD. One randomized cross-over RCT showed left-sided rTMS to be superior to the right [83], but with small sample sizes and only 5 treatment sessions for each condition; another small RCT showed no differences between right, left, or sham stimulation [84]. Notwithstanding the standard theoretical rationale of using LF-rTMS to the right DLPFC, HF-rTMS to this area remains relatively untested in MDD alone. Thus, for unilateral rTMS treatments, no single protocol is clearly efficacious for both MDD and AUD. However, we can hypothesize that excitatory stimulation of the DLPFC bilaterally is needed for an optimal effect when these conditions are comorbid, with amelioration of MDD symptoms from the left DLPFC and AUD symptoms from the right.

In line with this, for both MDD and AUD, dTMS using the H1 coil, which provides excitatory stimulation to the bilateral PFC more broadly, was shown to be effective. For MDD, this was confirmed through multiple large-scale, pivotal, double-blind RCTs [39], and for AUD through 2 pilot RCTs with positive findings [62,65]. Even more intriguing are open-label studies in patients with comorbid MDD + AUD showing a benefit from dTMS in both alcohol craving and depressive symptoms [64,70], and a non-randomized trial showing benefit in patients with comorbid dysthymic disorder and MDD [63]. To our knowledge, these are the only clinical trials of rTMS directly targeting the comorbidity of MDD + AUD. Thus, bilateral PFC stimulation either through sequential stimulation one side at a time or simultaneously through a dTMS coil (although worth noting that the most commonly studied H1 dTMS coil stimulates the left more than the right side) may be the most promising avenue for future study in comorbid MDD + AUD. Differences in dTMS coils are likely to be important, as shown by the lack of effect on AUD with the H8 coil that stimulated only the insula but spared the PFC.

To adequately develop rTMS for comorbid MDD + AUD, it will be important to adequately manage the additional safety concerns that come with problematic alcohol use. The most important of these is the increased risk of seizures, which is considered the most severe and potentially life-threatening adverse effect of rTMS. However, this is usually a rare event, with most estimations of seizure risk to be <0.01% [85,86]. Current safety guidelines for rTMS warn that alcohol abuse is a general risk factor for incident seizures and the state of alcohol withdrawal decreases the seizure threshold, and thus, it can be reasoned that AUD or general alcohol misuse is a risk factor for TMS-provoked seizures [30,85]. Despite this, these same guidelines do not explicitly state that patients with AUD should be excluded from rTMS, and simply recommend careful evaluation and documentation of alcohol use history and to “provide education” on the effects of alcohol use on rTMS. Currently, there is a lack of empirical evidence on the relative and absolute risk for seizures during rTMS in those with AUD, compared to those without AUD. One study of all documented seizures from dTMS identified through published case reports or reported to the manufacturer between 2010 and 2018 estimated an overall crude rate of 0.087% [87]. Of these, 6 out of 31 identified seizure cases related to increased alcohol use. These numbers suggest that even though alcohol can be a risk factor, the absolute risk is still exceedingly rare. These authors also pointed out that these seizure rates are better than those reported for common psychotropic medications that are commonly prescribed for patients, such as antidepressants and antipsychotics. Furthermore, given the well-known burden of illness from AUD in terms of increased morbidity and mortality, treatments that may be effective in ameliorating AUD but have a rare risk of an adverse event may still have a favorable risk-to-benefit ratio. Further research is also needed on how to design and modify treatment protocols to reduce the risk of seizures. Currently, all rTMS for AUD trials require participants to have safely completed medical detoxification and be out of the alcohol withdrawal period; however, details within these protocols vary greatly in terms of length of time after detox, length of abstinence before rTMS is started, and the treatment setting.

Another important area of exploration is how to develop novel therapeutics for comorbid conditions that are bidirectional in nature, as presentation can vary depending on the course of illness. For example, there is a large overlap between the presentation of alcohol-induced mood disorder and that of a primary mood disorder with comorbid AUD [88]. Diagnostic clarity often remains elusive, although a careful longitudinal history may provide clues [89]. Many patients with MDD can develop AUD after using alcohol to “self-medicate” their symptoms [90], and abstinence from alcohol can help alleviate symptoms of depression [91]. Thus, studies of rTMS for MDD + AUD should attempt to carefully distinguish and characterize the trajectory of illnesses in study participants to determine how the AUD and MDD symptoms interact and whether each, or both, of these conditions can be considered primary. Furthermore, on a neurobiological level, these conditions may not be discrete categories, and may lie on a continuum of psychopathology [92].

## 5. Neurobiologically Based Transdiagnostic Clinical Outcomes

The ability to directly modulate the neural circuits with rTMS is an exciting opportunity both within and across disorders. Transdiagnostic approaches to psychiatry have been favored in recent decades, given the well-described limitations of the current standards of diagnosis through the Diagnostic and Statistical Manual of Mental Disorders (DSM) related to excessive comorbidity between disorders and heterogeneity within disorders. This is evident through epidemiological studies showing extremely high rates of comorbidity. Genes, brain regions, and neural circuits implicated in the pathophysiology of various mental disorders have a high degree of overlap [93,94], suggesting a biological basis for comorbidity. For example, large-scale analyses of genome wide data from the Psychiatric Genomics Consortium and U.K. Biobank could demonstrate shared genetic mechanisms and a causal relationship of MDD on the development of AUD [95]. Furthermore, common neurobiological abnormalities between disorders may better map onto dysfunction in discrete behavioral functions or symptom clusters as opposed to entire syndromes. Researchers have been galvanized to this approach by the National Institutes of Mental Health’s Research Domain Criteria (RDoC), a novel classification framework that categorizes normal and pathological domains of brain function that are informed by neuroscience [96]. Here, we highlight the potential of rTMS to target transdiagnostic symptom constructs with high clinical relevance to both MDD and AUD, the cognitive system.

Cognitive dysfunction is understood to be a common and core symptom domain in both MDD and AUD, with executive dysfunction being among the most commonly reported and consistently significant findings [97], which include subdomains, such as working memory, inhibitory control, and set shifting [98]. Shared neurobiology may underlie this transdiagnostic symptom construct. One study showed that volumetric changes in the dorsal anterior cingulate and bilateral insula common across multiple diagnoses, including MDD and substance use disorders (SUDs), was associated with executive function deficits [94]. These regions interrelate with the DLPFC in order to balance between the cognitive focus of external tasks and salience processing [71]. Early TMS research in healthy control subjects were able to demonstrate cognitive enhancing changes, and that PFC stimulation affected executive functioning tasks, such as stimulus processing on the visual oddball task [99]. Given the weight of evidence in DLPFC stimulation using rTMS, the association of this region with executive function is important [100]. In MDD, the DLPFC has a key role in executive and cognitive control of negative emotion through reappraisal and suppression strategies [101]. In general, PFC subregions are interrelated and are involved in important functions related to salience detection, error prediction, and appraising the value of a given external stimulus [102,103]. These functions have wide clinical implications, such as on the processing of external- and internal-substance-related cues important in AUD, and for the physiological and psychological responses to safety and threat information important in mood and anxiety disorders [104]. The PFC’s role in cognition also extends to the integration of sensory and emotional signals from other parts of the brain [105] and in social cognition [106]. In AUD, activation of the DLPFC via rTMS may aid in modulating inhibitory processes that regulate risk-taking behavior and cravings [107]. This is in line with research showing the use of TMS in modulating action inhibition in humans as measured by their performance on the stop-signal task through stimulation of the DLPFC [108]. Importantly, emotional stimuli processing in the brain can also disrupt inhibition performance, pointing to a possible interaction effect between cognition and affective symptoms reported by patients [109]. Indeed, experimental studies have shown how priming by negative emotionality affects motor responses through increases or decreases in corticospinal excitability [110].

It is intriguing to note the parallel findings on the implications of executive dysfunction in MDD and AUD, such as greater treatment resistance and overall poorer outcome [111]. Furthermore, standard treatments may not directly address these deficits, given that studies of remitted MDD [112] and abstinent AUD patients [113] still have worse executive function, compared to healthy controls. For studies of DLPFC rTMS for MDD, a systematic review and meta-analysis by Martin and colleagues showed modest benefits in set shifting and psychomotor speed, two subcomponents of executive function [77]. Furthermore, the improvement from rTMS in executive function correlates well with depressed mood reduction [78]. In AUD, one study found promising results of HF-rTMS to the left DLPFC on set shifting and inhibition [60], and HF-rTMS to the right DLPFC resulted in more stable speed of response [53], but another study showed that it did not change performance on tasks of regulating impulsivity [68]. Cognitive improvements were not always associated with improvements on alcohol craving [60], and thus, the clinical relevance of cognitive task performance on clinical outcomes in AUD requires further study. Another study showed that HF-rTMS to the right DLPFC increased the frontal–parietal cognitive control network connectivity compared to sham, and that higher connectivity in this network was associated with fewer relapses [54]. The effect of rTMS is also transdiagnostic beyond MDD and AUD, with evidence existing in schizophrenia, bipolar disorder, and Alzheimer’s disease [114,115]. Still, the literature is currently limited by substantial variability and lack of consensus for rTMS protocols and target neurocognitive outcomes. Nonetheless, the effect of rTMS on enhancing cognition and treating executive dysfunction demonstrates the ability to target comorbidity broadly, including MDD + AUD comorbidity, if there exists an overlapping discrete symptom construct.

## 6. Future Directions to Optimize Efficacy of rTMS for MDD and AUD

An evolving area of research is the optimization and personalization of treatment. Although rTMS has proven efficacy in MDD, response and remission rates are argued to be modest, often attributed to inter-individual variability [45]. Novel treatment paradigms have thus been developed to address the questions of how to personalize treatment to the person and optimize effectiveness for more patients, and we highlight here some promising avenues that have potential for use in both MDD and AUD.

Considerable inter-individual heterogeneity in the structure of the head and brain anatomy of the DLPFC exists, which can influence the electric field induced in the brain by the magnetic field created by rTMS [116]. Scalp-based measurement may be imprecise in its ability to localize the target site of interest [117] and individualized MRI-guided “neuronavigation” techniques allow for more accurate positioning of the coil to stimulate group-based cortical targets [118]. However, even with this precision, there can be variability in the response, depending on sub-regions within the DLPFC [119], and evidence for superiority of neuronavigation compared to standard landmarking is mixed [79,120], leading to efforts for further optimization through functional connectivity between different areas of the DLPFC to other brain regions [80]. This has resulted in the finding that the region of the DLPFC that is the most anticorrelated with the subgenual anterior cingulate cortex (ACC) is associated with the greatest antidepressant effects, which was impressively replicated in a number of independent cohorts [81,121].

While this DLPFC and subgenual ACC circuit is considered important in MDD pathophysiology, in the study of AUD, Herremans et al. [57] hypothesized that because of the role of the ACC as a reward processing region implicated in alcohol relapse [122], it is a “relapse neurocircuit” with the DLPFC and ventromedial PFC (VMPFC) and could perhaps predict clinical outcomes from rTMS. They found that after HF-rTMS to the DLPFC, the lower the dorsal ACC activation at baseline, the more its activity increased after treatment [57]. Similarly, rTMS to the VMPFC/orbitofrontal cortex induced functional connectivity changes to several regions implicated in reward processing, among them the ACC, insula, and ventral striatum [66,67]. However, the clinical effects of rTMS to the MPFC have not been well established in MDD or AUD as yet. A promising strategy may lie in targeting functional connectivity between regions of the PFC connected to the ACC and related deeper structures implicated in reward processing.

Another approach to optimize rTMS outcomes is to increase the speed of response by condensing the course of treatment through accelerated rTMS. Instead of once-daily treatments, multiple sessions of rTMS per day are delivered over fewer days overall, and by optimally spacing sessions, there may be an enhanced effect through an accumulation of synaptic strengthening [123]. Feasibility and promising results for this method for MDD were demonstrated through open-label and sham-controlled trials, with high response/remission rates just after a few days of treatment [48,124]; however, findings of the superiority of accelerated rTMS over standard rTMS are still lacking [125]. In AUD, Herremans et al. [56] used 14 HF-rTMS treatments over 3 days, which demonstrated feasibility and effectiveness with an accelerated protocol. Follow-up studies with an expanded sample also used this same accelerated protocol [57,82] to examine neurobiological predictors but also continued to demonstrate feasibility. Importantly, in AUD, this accelerated protocol demonstrates safety in terms of seizure risk, given that the increasing number of pulses and sessions per day could theoretically confer a higher risk of seizures [30,85].

Lastly, a fascinating area of development in rTMS research, which could be applied to both MDD and AUD, is employing a task for participants to engage in, or a disorder-specific cue immediately before or during the stimulation. These protocols aim to exploit mechanisms of task-dependent plasticity, as once a disorder specific neurocircuit is “activated”, it may be more primed for neuromodulation by rTMS. For example, dTMS for the treatment of nicotine addiction is done with the pairing of smoking-related cues during treatment [73]. This combined method resulted in clinically significant findings within a multi-site RCT, which led to FDA approval. Indeed, across other SUDs, rTMS to treat cravings is commonly done in conjunction with a cue-induced paradigm [74]. For AUD, Ceccanti and colleagues [62] conducted a small sham-controlled RCT of dTMS administered immediately after participants were asked to look at and smell a glass filled with their favorite alcoholic drink, further individualizing the alcohol cue to patient preference. This may potentially result in more powerful engagement of the craving neurocircuit. Some single-session experimental studies also employed protocols asking participants to recall their most recent experience, using their substance of choice [66] with their rTMS treatment. For MDD, research is still exploring promising task- or cue-based paradigms. One avenue that was reported is the pairing of cognitive behavioral therapy with rTMS, based on the concept that there is a synergistic induction of neuroplasticity to cognitive control circuits [126]. However, a significant limitation of this current research is that there are no studies directly comparing rTMS with and without a behavioral cue; thus, the relative benefit of combining these treatments remains to be seen. For the treatment of patients with comorbid MDD + AUD, future research should inquire whether optimal treatment requires the addition of tasks activating the neurocircuitry underlying symptoms relevant to both disorders simultaneously or different tasks per disorder sequentially, and also to compare it with rTMS treatment alone.

## 7. Conclusions

In summary, the development of an effective intervention for patients with comorbid AUD + MDD remains a therapeutic challenge that has significant implications in decreasing morbidity risk and burden of illness. rTMS has proven efficacy, safety, and tolerability over several decades of research and integration into clinical care for MDD. The evidence for rTMS is well supported by numerous large-scale multi-site RCTs and federal regulatory approval for its use in clinical practice. For AUD, there is now approximately a decade of publications showing the potential of rTMS to reduce cravings and consumption. Currently, there is considerable variability in the methodological approach, with no clearly superior rTMS protocol for AUD established yet. We propose that synthesizing the converging points of evidence in these two bodies of literature can provide avenues for future research (Table 2), suggesting the considerable potential in developing rTMS for the treatment of individuals with these comorbid conditions. Our review suggests that rTMS for comorbid MDD + AUD may require a bilateral approach, given the strength of evidence of left and bilateral rTMS in MDD and of right and bilateral rTMS in AUD. It will also be important for future research to systematically examine the risk–benefit ratio of rTMS, given the risk of seizures associated with AUD. Exploring rTMS as a targeted therapy for transdiagnostic symptom domains guided by neurobiology, or through frameworks such as RDoC as opposed to DSM-based diagnoses, may also allow us to target comorbidity in a less constrained manner. There also appears to be high potential for novel rTMS protocols aimed at personalizing and optimizing treatment via neuroimaging guided targeting, accelerated rTMS, and combination with behavioral tasks or psychological cues. Ultimately, further research into rTMS for MDD + AUD brings hope for filling an unmet need for the patients suffering from this complex comorbidity.

## Figures and Tables

**Table 1:** Summary of clinical trials of rTMS for AUD.

Reference	*N* (Active, Control)	Frequency	# Sessions	Pulses/Session	Site	Coil	Clinical Outcome
Mishra (2010) [51]	30, 15	10 Hz	10	1000	R DLPFC	Figure-of-8	Decrease craving compared to sham
Höppner (2011) [59]	10, 9	20 Hz	10	1000	L DLPFC	Figure-of-8	No difference in craving or depressive symptoms compared to sham
Herremans (2012) [52]	15, 16	20 Hz	1	1560	R DLPFC	Figure-of-8	No difference in craving compared to sham
Herremans (2013) [53]	29, 29	20 Hz	2	1560	R DLPFC	Figure-of-8	No difference on craving compared to sham
Ceccanti (2015) [62]	9, 9	20 Hz	10	1000	BL MPFC	H-Coil	Decrease in craving and consumption compared to sham
Girardi (2015) [63]	10, 10	20 Hz	20	2200	Medial and Lateral PFC L > R	H-coil	Craving scores and depressive symptoms reduced compared to control group
Herremans (2015) [56]	26	20 Hz	15	1560	R DLPFC	Figure-of-8	General craving, but not cue induced craving, decreased
Mishra (2015) [61]	20	10 Hz	10	1000	R v. L DLPFC	Figure-of-8	Reduction in craving, no difference between R and L DLPFC
Rapinesi (2015) [64]	13	18 Hz	20	1980	DLPFC L > R	H-coil	Reduction in craving and depressive symptoms
Del Felice (2016) [60]	8, 9	10 Hz	4	1000	L DLPFC	Figure-of-8	No changes in craving and number of drinks
Addolorato (2017) [65]	5, 6	10 Hz	12	1000	L > R DLPFC	H-coil	Decreased number of drinking days compared to sham, no differences in cravings
Hanlon (2017) [66]	24, 24	cTBS	1	3600	VMPFC	Figure-of-8	No differences in craving compared to sham
Kearney-Ramos (2018) [67]	24, 24	cTBS	1	3600	VMPFC	Figure-of-8	No change in craving compared to sham
McNeill (2018) [58]	20	cTBS	1	600	R DLPFC	Figure-of-8	Ad libitum alcohol consumption in lab increased
Jansen (2019) [55]	39, 36	10 Hz	1	3000	R DLPFC	Figure-of-8	No difference in craving compared to sham
Schluter (2019) [68]	41, 41	10 Hz	10	3000	R DLPFC	Figure-of-8	No difference in impulsivity and inhibitory control compared to sham
Perini (2020) [69]	29, 27	10 Hz	15	1500	Insula	H-coil	No difference in craving and consumption compared to sham

R, right; L, left; DLPFC, dorsolateral prefrontal cortex; MPFC, medial prefrontal cortex; cTBS, continuous theta burst stimulation.

**Table 2:** Selected literature of converging directions for repetitive transcranial magnetic stimulation (rTMS) in major depressive disorder (MDD) and alcohol use disorder (AUD).

	MDD	AUD
rTMS Site/Frequency	HF Left DLPFC [15,30,40] LF Right DLPFC [15,30,40] HF Bilateral PFC ^1^ [15,30,40]	HF Right DLPFC [51,56,57] HF Bilateral PFC [62,63,64,65,70]
rTMS Coil	Figure-of-8 [15,30,40] Deep TMS [15,30,40]	Figure-of-8 [51,56,57,61] Deep TMS [62,63,64,65,70]
Adequate No. of Sessions	20–30 [15,30,40]	≥15 [56,63,64]
Transdiagnostic Symptom Construct	Executive function [77,78]	Executive function [53,60] Craving [74]
Optimization Protocols	Neuroimaging-guided targeting: anatomical (DLPFC) [79] and functional DLPFC to ACC [80,81] Accelerated rTMS [48]	Individualized effects on neuroimaging: ACC and reward circuit [57,66,67] Accelerated rTMS [56,57,82]

HF, high frequency; LF, low frequency; DLPFC, dorsolateral prefrontal cortex; PFC, prefrontal cortex; ACC, anterior cingulate cortex. ^1^ Using deep TMS coils, targets DLPFC but extends broadly across the PFC, including deeper structures below the PFC.

## References

[1] Grant B.F., Stinson F.S., Dawson D.A., Chou S.P., Dufour M.C., Compton W., Pickering R.P., Kaplan K. (2004). Prevalence and co-occurrence of substance use disorders and independent mood and anxiety disorders: Results from the National Epidemiologic Survey on Alcohol and Related Conditions. Arch. Gen. Psychiatry.

[2] Hasin D.S., Goodwin R.D., Stinson F.S., Grant B.F. (2005). Epidemiology of major depressive disorder: Results from the National Epidemiologic Survey on Alcoholism and Related Conditions. Arch. Gen. Psychiatry.

[3] Kessler R.C., Borges G., Walters E.E. (1999). Prevalence of and risk factors for lifetime suicide attempts in the National Comorbidity Survey. Arch. Gen. Psychiatry.

[4] Davis L.L., Wisniewski S.R., Howland R.H., Trivedi M.H., Husain M.M., Fava M., McGrath P.J., Balasubramani G.K., Warden D., Rush A.J. (2010). Does comorbid substance use disorder impair recovery from major depression with SSRI treatment? An analysis of the STAR*D level one treatment outcomes. Drug Alcohol Depend..

[5] Sanchez K., Walker R., Campbell A.N., Greer T.L., Hu M.C., Grannemann B.D., Nunes E.V., Trivedi M.H. (2015). Depressive symptoms and associated clinical characteristics in outpatients seeking community-based treatment for alcohol and drug problems. Subst. Abus..

[6] Fortney J.C., Booth B.M., Curran G.M. (1999). Do patients with alcohol dependence use more services? A comparative analysis with other chronic disorders. Alcohol Clin. Exp. Res..

[7] Agabio R., Trogu E., Pani P.P. (2018). Antidepressants for the treatment of people with co-occurring depression and alcohol dependence. Cochrane Database Syst Rev..

[8] Foulds J.A., Adamson S.J., Boden J.M., Williman J.A., Mulder R.T. (2015). Depression in patients with alcohol use disorders: Systematic review and meta-analysis of outcomes for independent and substance-induced disorders. J. Affect. Disord..

[9] Stokes P.R.A., Jokinen T., Amawi S., Qureshi M., Husain M.I., Yatham L.N., Strang J., Young A.H. (2020). Pharmacological treatment of mood disorders and comorbid addictions: A systematic review and meta-analysis. Traitement pharmacologique des troubles de l’humeur et des dépendances comorbides: Une revue systématique et une méta-analyse. Can. J. Psychiatry.

[10] Adamson S.J., Sellman J.D., Foulds J.A., Frampton C.M., Deering D., Dunn A., Berks J., Nixon L., Cape G. (2015). A randomized trial of combined citalopram and naltrexone for nonabstinent outpatients with co-occurring alcohol dependence and major depression. J. Clin. Psychopharmacol..

[11] Oslin D.W. (2005). Treatment of late-life depression complicated by alcohol dependence. Am. J. Geriatr. Psychiatry.

[12] Pettinati H.M., Oslin D.W., Kampman K.M., Dundon W.D., Xie H., Gallis T.L., Dackis C.A., O’Brien C.P. (2010). A double-blind, placebo-controlled trial combining sertraline and naltrexone for treating co-occurring depression and alcohol dependence. Am. J. Psychiatry.

[13] DeVido J.J., Weiss R.D. (2012). Treatment of the depressed alcoholic patient. Curr. Psychiatry Rep..

[14] Hakobyan S., Vazirian S., Lee-Cheong S., Krausz M., Honer W.G., Schutz C.G. (2020). Concurrent disorder management guidelines. Systematic review. J. Clin. Med..

[15] Lefaucheur J.P., Aleman A., Baeken C., Benninger D.H., Brunelin J., di Lazzaro V., Filipovic S.R., Grefkes C., Hasan A., Hummel F.C. (2020). Evidence-based guidelines on the therapeutic use of repetitive transcranial magnetic stimulation (rTMS): An update (2014–2018). Clin. Neurophysiol..

[16] Deng Z.D., Lisanby S.H., Peterchev A.V. (2013). Electric field depth-focality tradeoff in transcranial magnetic stimulation: Simulation comparison of 50 coil designs. Brain Stimul..

[17] Kim H.K., Blumberger D.M., Downar J., Daskalakis Z.J. (2021). Systematic review of biological markers of therapeutic repetitive transcranial magnetic stimulation in neurological and psychiatric disorders. Clin. Neurophysiol..

[18] Tik M., Hoffmann A., Sladky R., Tomova L., Hummer A., Navarro de Lara L., Bukowski H., Pripfl J., Biswal B., Lamm C. (2017). Towards understanding rTMS mechanism of action: Stimulation of the DLPFC causes network-specific increase in functional connectivity. Neuroimage.

[19] Borgomaneri S., Battaglia S., Garofalo S., Tortora F., Avenanti A., di Pellegrino G. (2020). State-dependent TMS over prefrontal cortex disrupts fear-memory reconsolidation and prevents the return of fear. Curr. Biol..

[20] Borgomaneri S., Battaglia S., Avenanti A., Pellegrino G.D. (2021). Don’t hurt me no more: State-dependent transcranial magnetic stimulation for the treatment of specific phobia. J. Affect. Disord..

[21] Borgomaneri S., Battaglia S., Sciamanna G., Tortora F., Laricchiuta D. (2021). Memories are not written in stone: Re-writing fear memories by means of non-invasive brain stimulation and optogenetic manipulations. Neurosci. Biobehav. Rev..

[22] Carpenter L.L., Janicak P.G., Aaronson S.T., Boyadjis T., Brock D.G., Cook I.A., Dunner D.L., Lanocha K., Solvason H.B., Demitrack M.A. (2012). Transcranial magnetic stimulation (TMS) for major depression: A multisite, naturalistic, observational study of acute treatment outcomes in clinical practice. Depress. Anxiety.

[23] Somani A., Kar S.K. (2019). Efficacy of repetitive transcranial magnetic stimulation in treatment-resistant depression: The evidence thus far. Gen. Psychiatr..

[24] Elias G.J.B., Boutet A., Parmar R., Wong E.H.Y., Germann J., Loh A., Paff M., Pancholi A., Gwun D., Chow C.T. (2021). Neuromodulatory treatments for psychiatric disease: A comprehensive survey of the clinical trial landscape. Brain Stimul..

[25] Höflich G., Kasper S., Hufnagel A., Ruhrmann S., Möller H.J. (1993). Application of transcranial magnetic stimulation in treatment of drug-resistant major depression—A report of two cases. Hum. Psychopharmacol. Clin. Experimental..

[26] Belmaker R.H., Fleischmann A. (1995). Transcranial magnetic stimulation: A potential new frontier in Psychiatry. Biol. Psychiatry.

[27] George M.S., Wassermann E.M., Williams W.A., Callahan A., Ketter T.A., Basser P., Hallet M., Post R.M. (1995). Daily repetitive transcranial magnetic stimulation (rTMS) improves mood in depression. Neuroreport.

[28] George M.S., Lisanby S.H., Avery D., McDonald W.M., Durkalski V., Pavlicova M., Anderson B., Nahas Z., Bulow P., Zarkowski P. (2010). Daily left prefrontal transcranial magnetic stimulation therapy for major depressive disorder: A sham-controlled randomized trial. Arch. Gen. Psychiatry.

[29] O’Reardon J.P., Solvason H.B., Janicak P.G., Sampson S., Isenberg K.E., Nahas Z., McDonald W.M., Avery D., Fitzgerald P.B., Loo C. (2007). Efficacy and safety of transcranial magnetic stimulation in the acute treatment of major depression: A multisite randomized controlled trial. Biol. Psychiatry.

[30] McClintock S.M., Reti I.M., Carpenter L.L., McDonald W.M., Dubin M., Taylor S.F., Cook I.A., O’Reardon J., Husain M.M., Wall C. (2018). Consensus recommendations for the clinical application of repetitive transcranial magnetic stimulation (rTMS) in the treatment of depression. J. Clin. Psychiatry.

[31] Garcia-Toro M., Montes J.M., Talavera J.A. (2001). Functional cerebral asymmetry in affective disorders: New facts contributed by transcranial magnetic stimulation. J. Affect. Disord..

[32] Speer A.M., Benson B.E., Kimbrell T.K., Wassermann E.M., Willis M.W., Herscovitch P., Post R.M. (2009). Opposite effects of high and low frequency rTMS on mood in depressed patients: Relationship to baseline cerebral activity on PET. J. Affect. Disord..

[33] Brunoni A.R., Chaimani A., Moffa A.H., Razza L.B., Gattaz W.F., Daskalakis Z.J., Carvalho A.F. (2017). Repetitive transcranial magnetic stimulation for the acute treatment of major depressive episodes: A systematic review with network meta-analysis. JAMA Psychiatry.

[34] Fitzgerald P.B., Brown T.L., Marston N.A., Daskalakis Z.J., de Castella A., Kulkarni J. (2003). Transcranial magnetic stimulation in the treatment of depression: A double-blind, placebo-controlled trial. Arch. Gen. Psychiatry.

[35] Berlim M.T., van den Eynde F., Daskalakis Z.J. (2013). Efficacy and acceptability of high frequency repetitive transcranial magnetic stimulation (rTMS) versus electroconvulsive therapy (ECT) for major depression: A systematic review and meta-analysis of randomized trials. Depress. Anxiety.

[36] Roth Y., Amir A., Levkovitz Y., Zangen A. (2007). Three-dimensional distribution of the electric field induced in the brain by transcranial magnetic stimulation using figure-8 and deep H-coils. J. Clin. Neurophysiol..

[37] Parazzini M., Fiocchi S., Chiaramello E., Roth Y., Zangen A., Ravazzani P. (2017). Electric field estimation of deep transcranial magnetic stimulation clinically used for the treatment of neuropsychiatric disorders in anatomical head models. Med. Eng. Phys..

[38] Gersner R., Toth E., Isserles M., Zangen A. (2010). Site-specific antidepressant effects of repeated subconvulsive electrical stimulation: Potential role of brain-derived neurotrophic factor. Biol. Psychiatry.

[39] Levkovitz Y., Isserles M., Padberg F., Lisanby S.H., Bystritsky A., Xia G., Tendler A., Daskalakis Z.J., Winston J.L., Dannon P. (2015). Efficacy and safety of deep transcranial magnetic stimulation for major depression: A prospective multicenter randomized controlled trial. World Psychiatry.

[40] Milev R.V., Giacobbe P., Kennedy S.H., Blumberger D.M., Daskalakis Z.J., Downar J., Modirrousta M., Patry S., Vila-Rodriguez F., Lam R.W. (2016). Canadian network for mood and anxiety treatments (CANMAT) 2016 clinical guidelines for the management of adults with major depressive disorder: Section 4. Neurostimulation treatments. Can. J. Psychiatry.

[41] Sehatzadeh S., Daskalakis Z.J., Yap B., Tu H.A., Palimaka S., Bowen J.M., O’Reilly D.J. (2019). Unilateral and bilateral repetitive transcranial magnetic stimulation for treatment-resistant depression: A meta-analysis of randomized controlled trials over 2 decades. J. Psychiatry Neurosci..

[42] Huang Y.Z., Rothwell J.C., Chen R.S., Lu C.S., Chuang W.L. (2011). The theoretical model of theta burst form of repetitive transcranial magnetic stimulation. Clin. Neurophysiol..

[43] Huang Y.-Z., Edwards M.J., Rounis E., Bhatia K.P., Rothwell J.C. (2005). Theta burst stimulation of the human motor cortex. Neuron.

[44] Blumberger D.M., Vila-Rodriguez F., Thorpe K.E., Feffer K., Noda Y., Giacobbe P., Knyahnytska Y., Kennedy S.H., Lam R.W., Daskalakis Z.J. (2018). Effectiveness of theta burst versus high-frequency repetitive transcranial magnetic stimulation in patients with depression (THREE-D): A randomised non-inferiority trial. Lancet.

[45] Miron J.P., Jodoin V.D., Lespérance P., Blumberger D.M. (2021). Repetitive transcranial magnetic stimulation for major depressive disorder: Basic principles and future directions. Ther Adv. Psychopharmacol..

[46] Bakker N., Shahab S., Giacobbe P., Blumberger D.M., Daskalakis Z.J., Kennedy S.H., Downar J. (2015). rTMS of the dorsomedial prefrontal cortex for major depression: Safety, tolerability, effectiveness, and outcome predictors for 10 Hz versus intermittent theta-burst stimulation. Brain Stimul..

[47] Fitzgerald P.B., Hoy K., McQueen S., Herring S., Segrave R., Been G., Kulkarni J., Daskalakis Z.J. (2008). Priming stimulation enhances the effectiveness of low-frequency right prefrontal cortex transcranial magnetic stimulation in major depression. J. Clin. Psychopharmacol..

[48] Cole E.J., Phillips A.L., Bentzley B.S., Stimpson K.H., Nejad R., Barmak F., Veerapal C., Khan N., Cherian K., Felber E. (2021). Stanford Neuromodulation Therapy (SNT): A double-blind randomized controlled trial. Am. J. Psychiatry.

[49] Eichhammer P., Johann M., Kharraz A., Binder H., Pittrow D., Wodarz N., Hajak G. (2003). High-frequency repetitive transcranial magnetic stimulation decreases cigarette smoking. J. Clin. Psychiatry.

[50] Camprodon J.A., Martínez-Raga J., Alonso-Alonso M., Shih M.C., Pascual-Leone A. (2007). One session of high frequency repetitive transcranial magnetic stimulation (rTMS) to the right prefrontal cortex transiently reduces cocaine craving. Drug Alcohol Depend..

[51] Mishra B.R., Nizamie S.H., Das B., Praharaj S.K. (2010). Efficacy of repetitive transcranial magnetic stimulation in alcohol dependence: A sham-controlled study. Addiction.

[52] Herremans S.C., Baeken C., Vanderbruggen N., Vanderhasselt M.A., Zeeuws D., Santermans L., De Raedt R. (2012). No influence of one right-sided prefrontal HF-rTMS session on alcohol craving in recently detoxified alcohol-dependent patients: Results of a naturalistic study. Drug Alcohol Depend..

[53] Herremans S.C., Vanderhasselt M.A., de Raedt R., Baeken C. (2013). Reduced intra-individual reaction time variability during a Go-NoGo task in detoxified alcohol-dependent patients after one right-sided dorsolateral prefrontal HF-rTMS session. Alcohol Alcohol..

[54] Jansen J.M., van Wingen G., van den Brink W., Goudriaan A.E. (2015). Resting state connectivity in alcohol dependent patients and the effect of repetitive transcranial magnetic stimulation. Eur. Neuropsychopharmacol..

[55] Jansen J.M., van den Heuvel O.A., van der Werf Y.D., de Wit S.J., Veltman D.J., van den Brink W., Goudriaan A.E. (2019). The effect of high-frequency repetitive transcranial magnetic stimulation on emotion processing, reappraisal, and craving in alcohol use disorder patients and healthy controls: A functional magnetic resonance imaging study. Front. Psychiatry.

[56] Herremans S.C., Van Schuerbeek P., de Raedt R., Matthys F., Buyl R., de Mey J., Baeken C. (2015). The Impact of accelerated right prefrontal high-frequency repetitive transcranial magnetic stimulation (rTMS) on cue-reactivity: An fMRI study on craving in recently detoxified alcohol-dependent patients. PLoS ONE.

[57] Herremans S.C., de Raedt R., van Schuerbeek P., Marinazzo D., Matthys F., de Mey J., Baeken C. (2016). Accelerated HF-rTMS protocol has a rate-dependent effect on dacc activation in alcohol-dependent patients: An open-label feasibility study. Alcohol Clin. Exp. Res..

[58] McNeill A., Monk R.L., Qureshi A.W., Makris S., Heim D. (2018). Continuous Theta burst transcranial magnetic stimulation of the right dorsolateral prefrontal cortex impairs inhibitory control and increases alcohol consumption. Cogn. Affect. Behav. Neurosci..

[59] Höppner J., Broese T., Wendler L., Berger C., Thome J. (2011). Repetitive transcranial magnetic stimulation (rTMS) for treatment of alcohol dependence. World J. Biol. Psychiatry.

[60] Del Felice A., Bellamoli E., Formaggio E., Manganotti P., Masiero S., Cuoghi G., Rimondo C., Genetti B., Sperotto M., Corso F. (2016). Neurophysiological, psychological and behavioural correlates of rTMS treatment in alcohol dependence. Drug Alcohol Depend..

[61] Mishra B.R., Praharaj S.K., Katshu M.Z., Sarkar S., Nizamie S.H. (2015). Comparison of anticraving efficacy of right and left repetitive transcranial magnetic stimulation in alcohol dependence: A randomized double-blind study. J. Neuropsychiatry Clin. Neurosci..

[62] Ceccanti M., Inghilleri M., Attilia M.L., Raccah R., Fiore M., Zangen A. (2015). Deep TMS on alcoholics: Effects on cortisolemia and dopamine pathway modulation. A pilot study. Can. J. Physiol Pharmacol..

[63] Girardi P., Rapinesi C., Chiarotti F., Kotzalidis G.D., Piacentino D., Serata D., Del Casale A., Scatena P., Mascioli F., Raccah R.N. (2015). Add-on deep transcranial magnetic stimulation (dTMS) in patients with dysthymic disorder comorbid with alcohol use disorder: A comparison with standard treatment. World J. Biol. Psychiatry.

[64] Rapinesi C., Curto M., Kotzalidis G.D., del Casale A., Serata D., Ferri V.R., Di Pietro S., Scatena P., Bersani F.S., Raccah R.N. (2015). Antidepressant effectiveness of deep Transcranial Magnetic Stimulation (dTMS) in patients with Major Depressive Disorder (MDD) with or without Alcohol Use Disorders (AUDs): A 6-month, open label, follow-up study. J. Affect. Disord..

[65] Addolorato G., Antonelli M., Cocciolillo F., Vassallo G.A., Tarli C., Sestito L., Mirijello A., Ferrulli A., Pizzuto D.A., Camardese G. (2017). Deep transcranial magnetic stimulation of the dorsolateral prefrontal cortex in alcohol use disorder patients: Effects on dopamine transporter availability and alcohol intake. Eur. Neuropsychopharmacol..

[66] Hanlon C.A., Dowdle L.T., Correia B., Mithoefer O., Kearney-Ramos T., Lench D., Griffin M., Anton R.F., George M.S. (2017). Left frontal pole theta burst stimulation decreases orbitofrontal and insula activity in cocaine users and alcohol users. Drug Alcohol Depend..

[67] Kearney-Ramos T.E., Dowdle L.T., Lench D.H., Mithoefer O.J., Devries W.H., George M.S., Anton R.F., Hanlon C.A. (2018). Transdiagnostic effects of ventromedial prefrontal cortex transcranial magnetic stimulation on cue reactivity. Biol. Psychiatry Cogn. Neurosci. Neuroimaging.

[68] Schluter R.S., van Holst R.J., Goudriaan A.E. (2019). Effects of ten sessions of high frequency repetitive transcranial magnetic stimulation (HF-rTMS) add-on treatment on impulsivity in alcohol use disorder. Front. Neurosci..

[69] Perini I., Kämpe R., Arlestig T., Karlsson H., Löfberg A., Pietrzak M., Zangen A., Heilig M. (2020). Repetitive transcranial magnetic stimulation targeting the insular cortex for reduction of heavy drinking in treatment-seeking alcohol-dependent subjects: A randomized controlled trial. Neuropsychopharmacology.

[70] Rapinesi C., Kotzalidis G.D., Ferracuti S., Girardi N., Zangen A., Sani G., Raccah R.N., Girardi P., Pompili M., Del Casale A. (2018). Add-on high frequency deep transcranial magnetic stimulation (dTMS) to bilateral prefrontal cortex in depressive episodes of patients with major depressive disorder, bipolar disorder I, and major depressive with alcohol use disorders. Neurosci Lett..

[71] Ibrahim C., Rubin-Kahana D.S., Pushparaj A., Musiol M., Blumberger D.M., Daskalakis Z.J., Zangen A., Le Foll B. (2019). The Insula: A brain stimulation target for the treatment of addiction. Front. Pharmacol..

[72] Dinur-Klein L., Dannon P., Hadar A., Rosenberg O., Roth Y., Kotler M., Zangen A. (2014). Smoking cessation induced by deep repetitive transcranial magnetic stimulation of the prefrontal and insular cortices: A prospective, randomized controlled trial. Biol. Psychiatry.

[73] Zangen A., Moshe H., Martinez D., Barnea-Ygael N., Vapnik T., Bystritsky A., Duffy W., Toder D., Casuto L., Grosz M.L. (2021). Repetitive transcranial magnetic stimulation for smoking cessation: A pivotal multicenter double-blind randomized controlled trial. World Psychiatry.

[74] Zhang J.J.Q., Fong K.N.K., Ouyang R.G., Siu A.M.H., Kranz G.S. (2019). Effects of repetitive transcranial magnetic stimulation (rTMS) on craving and substance consumption in patients with substance dependence: A systematic review and meta-analysis. Addiction.

[75] Teng S., Guo Z., Peng H., Xing G., Chen H., He B., McClure M.A., Mu Q. (2017). High-frequency repetitive transcranial magnetic stimulation over the left DLPFC for major depression: Session-dependent efficacy: A meta-analysis. Eur. Psychiatry.

[76] Shen Y., Cao X., Tan T., Shan C., Wang Y., Pan J., He H., Yuan T.-F. (2016). 10-Hz repetitive transcranial magnetic stimulation of the left dorsolateral prefrontal cortex reduces heroin cue craving in long-term addicts. Biol. Psychiatry.

[77] Martin D.M., McClintock S.M., Forster J.J., Lo T.Y., Loo C.K. (2017). Cognitive enhancing effects of rTMS administered to the prefrontal cortex in patients with depression: A systematic review and meta-analysis of individual task effects. Depress. Anxiety.

[78] Ilieva I.P., Alexopoulos G.S., Dubin M.J., Morimoto S.S., Victoria L.W., Gunning F.M. (2018). Age-related repetitive transcranial magnetic stimulation effects on executive function in depression: A systematic review. Am. J. Geriatr. Psychiatry.

[79] Fitzgerald P.B., Hoy K., McQueen S., Maller J.J., Herring S., Segrave R., Bailey M., Been G., Kulkarni J., Daskalakis Z.J. (2009). A randomized trial of rTMS targeted with MRI based neuro-navigation in treatment-resistant depression. Neuropsychopharmacology.

[80] Fox M.D., Liu H., Pascual-Leone A. (2013). Identification of reproducible individualized targets for treatment of depression with TMS based on intrinsic connectivity. Neuroimage.

[81] Weigand A., Horn A., Caballero R., Cooke D., Stern A.P., Taylor S.F., Press D., Pascual-Leone A., Fox M.D. (2018). Prospective validation that subgenual connectivity predicts antidepressant efficacy of transcranial magnetic stimulation sites. Biol. Psychiatry.

[82] Wu G.-R., Baeken C., van Schuerbeek P., de Mey J., Bi M., Herremans S.C. (2018). Accelerated repetitive transcranial magnetic stimulation does not influence grey matter volumes in regions related to alcohol relapse: An open-label exploratory study. Drug Alcohol Depend..

[83] Pascual-Leone A., Rubio B., Pallardó F., Catalá M.D. (1996). Rapid-rate transcranial magnetic stimulation of left dorsolateral prefrontal cortex in drug-resistant depression. Lancet.

[84] Triggs W.J., Ricciuti N., Ward H.E., Cheng J., Bowers D., Goodman W.K., Kluger B.M., Nadeau S.E. (2010). Right and left dorsolateral pre-frontal rTMS treatment of refractory depression: A randomized, sham-controlled trial. Psychiatry Res..

[85] Rossi S., Antal A., Bestmann S., Bikson M., Brewer C., Brockmöller J., Carpenter L.L., Cincotta M., Chen R., Daskalakis Z.J. (2021). Safety and recommendations for TMS use in healthy subjects and patient populations, with updates on training, ethical and regulatory issues: *Expert Guidelines*. Clin. Neurophysiol..

[86] Stultz D.J., Osburn S., Burns T., Pawlowska-Wajswol S., Walton R. (2020). Transcranial magnetic stimulation (TMS) safety with respect to seizures: A literature review. Neuropsychiatr. Dis. Treat..

[87] Tendler A., Roth Y., Zangen A. (2018). Rate of inadvertently induced seizures with deep repetitive transcranial magnetic stimulation. Brain Stimul..

[88] Schuckit M.A., Tipp J.E., Bergman M., Reich W., Hesselbrock V.M., Smith T.L. (1997). Comparison of induced and independent major depressive disorders in 2945 alcoholics. Am. J. Psychiatry.

[89] Hassan A.N. (2018). Patients with alcohol use disorder co-occurring with depression and anxiety symptoms: Diagnostic and treatment initiation recommendations. J. Clin. Psychiatry.

[90] Crum R.M., Mojtabai R., Lazareck S., Bolton J.M., Robinson J., Sareen J., Green K.M., Stuart E.A., La Flair L., Alvanzo A.A.H. (2013). A prospective assessment of reports of drinking to self-medicate mood symptoms with the incidence and persistence of alcohol dependence. JAMA Psychiatry.

[91] Brown S.A., Inaba R.K., Gillin J.C., Schuckit M.A., Stewart M.A., Irwin M.R. (1995). Alcoholism and affective disorder: Clinical course of depressive symptoms. Am. J. Psychiatry.

[92] Ng Q.X., Lim D.Y., Chee K.T. (2020). Reimagining the spectrum of affective disorders. Bipolar Disord..

[93] Solovieff N., Cotsapas C., Lee P.H., Purcell S.M., Smoller J.W. (2013). Pleiotropy in complex traits: Challenges and strategies. Nat. Rev. Genet..

[94] Goodkind M., Eickhoff S.B., Oathes D.J., Jiang Y., Chang A., Jones-Hagata L.B., Ortega B.N., Zaiko Y.V., Roach E.L., Korgaonkar M.S. (2015). Identification of a common neurobiological substrate for mental illness. JAMA Psychiatry.

[95] Polimanti R., Peterson R.E., Ong J.S., MacGregor S., Edwards A.C., Clarke T.K., Frank J., Gerring Z., Gillespie N.A., Lind P.A. (2019). Evidence of causal effect of major depression on alcohol dependence: Findings from the psychiatric genomics consortium. Psychol Med..

[96] Insel T., Cuthbert B., Garvey M., Heinssen R., Pine D.S., Quinn K., Sanislow C., Wang P. (2010). Research domain criteria (RDoC): Toward a new classification framework for research on mental disorders. Am. J. Psychiatry.

[97] Nuño L., Gómez-Benito J., Carmona V.R., Pino O. (2021). A systematic review of executive function and information processing speed in major depression disorder. Brain Sci..

[98] Jurado M.B., Rosselli M. (2007). The elusive nature of executive functions: A review of our current understanding. Neuropsychol. Rev..

[99] Evers S., Böckermann I., Nyhuis P.W. (2001). The impact of transcranial magnetic stimulation on cognitive processing: An event-related potential study. Neuroreport.

[100] Ridderinkhof K.R., van den Wildenberg W.P., Segalowitz S.J., Carter C.S. (2004). Neurocognitive mechanisms of cognitive control: The role of prefrontal cortex in action selection, response inhibition, performance monitoring, and reward-based learning. Brain Cogn..

[101] Koenigs M., Grafman J. (2009). The functional neuroanatomy of depression: Distinct roles for ventromedial and dorsolateral prefrontal cortex. Behav. Brain Res..

[102] Garofalo S., Timmermann C., Battaglia S., Maier M.E., di Pellegrino G. (2017). Mediofrontal Negativity signals unexpected timing of salient outcomes. J. Cogn Neurosci..

[103] Battaglia S., Garofalo S., di Pellegrino G., Starita F. (2020). Revaluing the role of vmPFC in the acquisition of pavlovian threat conditioning in humans. J. Neurosci..

[104] Battaglia S., Harrison B.J., Fullana M.A. (2021). Does the human ventromedial prefrontal cortex support fear learning, fear extinction or both? A commentary on subregional contributions. Mol. Psychiatry.

[105] Gilmartin M.R., Balderston N.L., Helmstetter F.J. (2014). Prefrontal cortical regulation of fear learning. Trends Neurosci..

[106] Hiser J., Koenigs M. (2018). The multifaceted role of the ventromedial prefrontal cortex in emotion, decision making, social cognition, and psychopathology. Biol. Psychiatry.

[107] Goldstein R.Z., Volkow N.D. (2011). Dysfunction of the prefrontal cortex in addiction: Neuroimaging findings and clinical implications. Nat. Rev. Neurosci..

[108] Borgomaneri S., Serio G., Battaglia S. (2020). Please, don’t do it! Fifteen years of progress of non-invasive brain stimulation in action inhibition. Cortex.

[109] Battaglia S., Serio G., Scarpazza C., D’Ausilio A., Borgomaneri S. (2021). Frozen in (e)motion: How reactive motor inhibition is influenced by the emotional content of stimuli in healthy and psychiatric populations. Behav Res. Ther..

[110] Borgomaneri S., Vitale F., Battaglia S., Avenanti A. (2021). Early right motor cortex response to happy and fearful facial expressions: A TMS motor-evoked potential study. Brain Sci..

[111] Jaeger J., Berns S., Uzelac S., Davis-Conway S. (2006). Neurocognitive deficits and disability in major depressive disorder. Psychiatry Res..

[112] Nakano Y., Baba H., Maeshima H., Kitajima A., Sakai Y., Baba K., Suzuki T., Mimura M., Arai H. (2008). Executive dysfunction in medicated, remitted state of major depression. J. Affect. Disord..

[113] Kopera M., Wojnar M., Brower K., Glass J., Nowosad I., Gmaj B., Szelenberger W. (2012). Cognitive functions in abstinent alcohol-dependent patients. Alcohol.

[114] Sciortino D., Pigoni A., Delvecchio G., Maggioni E., Schiena G., Brambilla P. (2021). Role of rTMS in the treatment of cognitive impairments in bipolar disorder and schizophrenia: A review of randomized controlled trials. J. Affect. Disord..

[115] Iimori T., Nakajima S., Miyazaki T., Tarumi R., Ogyu K., Wada M., Tsugawa S., Masuda F., Daskalakis Z.J., Blumberger D.M. (2019). Effectiveness of the prefrontal repetitive transcranial magnetic stimulation on cognitive profiles in depression, schizophrenia, and Alzheimer’s disease: A systematic review. Prog. Neuropsychopharmacol. Biol. Psychiatry.

[116] Peterchev A.V., Wagner T.A., Miranda P.C., Nitsche M.A., Paulus W., Lisanby S.H., Pascual-Leone A., Bikson M. (2012). Fundamentals of transcranial electric and magnetic stimulation dose: Definition, selection, and reporting practices. Brain Stimul..

[117] Herwig U., Padberg F., Unger J., Spitzer M., Schönfeldt-Lecuona C. (2001). Transcranial magnetic stimulation in therapy studies: Examination of the reliability of “standard” coil positioning by neuronavigation. Biol. Psychiatry.

[118] Blumberger D.M., Maller J.J., Thomson L., Mulsant B.H., Rajji T.K., Maher M., Brown P.E., Downar J., Vila-Rodriguez F., Fitzgerald P.B. (2016). Unilateral and bilateral MRI-targeted repetitive transcranial magnetic stimulation for treatment-resistant depression: A randomized controlled study. J. Psychiatry Neurosci..

[119] Herbsman T., Avery D., Ramsey D., Holtzheimer P., Wadjik C., Hardaway F., Haynor D., George M.S., Nahas Z. (2009). More lateral and anterior prefrontal coil location is associated with better repetitive transcranial magnetic stimulation antidepressant response. Biol. Psychiatry.

[120] Hebel T., Göllnitz A., Schoisswohl S., Weber F.C., Abdelnaim M., Wetter T.C., Rupprecht R., Langguth B., Schecklmann M. (2021). A direct comparison of neuronavigated and non-neuronavigated intermittent theta burst stimulation in the treatment of depression. Brain Stimul..

[121] Cash R.F.H., Cocchi L., Lv J., Fitzgerald P.B., Zalesky A. (2021). Functional magnetic resonance imaging-guided personalization of transcranial magnetic stimulation treatment for depression. JAMA Psychiatry.

[122] Volkow N.D., Boyle M. (2018). Neuroscience of addiction: Relevance to prevention and treatment. Am. J. Psychiatry.

[123] Goldsworthy M.R., Pitcher J.B., Ridding M.C. (2012). The application of spaced theta burst protocols induces long-lasting neuroplastic changes in the human motor cortex. Eur. J. Neurosci..

[124] McGirr A., van den Eynde F., Tovar-Perdomo S., Fleck M.P., Berlim M.T. (2015). Effectiveness and acceptability of accelerated repetitive transcranial magnetic stimulation (rTMS) for treatment-resistant major depressive disorder: An open label trial. J. Affect. Disord..

[125] Blumberger D.M., Vila-Rodriguez F., Wang W., Knyahnytska Y., Butterfield M., Noda Y., Yariv S., Isserles M., Voineskos D., Ainsworth N.J. (2021). A randomized sham controlled comparison of once vs twice-daily intermittent theta burst stimulation in depression: A Canadian rTMS treatment and biomarker network in depression (CARTBIND) study. Brain Stimul..

[126] Donse L., Padberg F., Sack A.T., Rush A.J., Arns M. (2018). Simultaneous rTMS and psychotherapy in major depressive disorder: Clinical outcomes and predictors from a large naturalistic study. Brain Stimul..

